# Targeting phosphofructokinase in cancer: integrating natural products for metabolic reprogramming and therapeutic innovation

**DOI:** 10.3389/fphar.2026.1844992

**Published:** 2026-06-10

**Authors:** Chengjian Cao, Chaoxiang Lv, Ali ElFar

**Affiliations:** 1 Zigong Academy of Medical Sciences, Zigong First People’s Hospital, Zigong, China; 2 Key Laboratory of Epigenetics and Oncology, The Research Center for Preclinical Medicine, Southwest Medical University, Luzhou, China

**Keywords:** cancer metabolism, metabolic reprogramming, PFK inhibitors, phosphofructokinase, therapeutic target, Warburg effect

## Abstract

The metabolism of cancer cells is reprogrammed toward aerobic glycolysis (the Warburg effect), which stimulates tumor growth. Phosphofructokinase (PFK) and its isoforms, PFKP, PFKM, and PFKL, are highly conserved central glycolytic controllers and a potential therapeutic intervention. This review discusses the complex functions of PFK in tumor biology, including its roles in regulating proliferation, invasion, metastasis, and therapy resistance. It further discusses the tumor microenvironmental role of PFK, which influences immune evasion, angiogenesis, and stromal interactions, as well as its non-metabolic signaling functions. The therapeutic approaches to PFK, such as synthetic (e.g., PFK15) and natural (e.g., curcumin) compounds, are considered alongside strategies to address specific difficulties. Lastly, the review is based on a combination of expression analysis of PFK isoforms and a closer analysis of synthetic and natural inhibitors, and it suggests a prospective framework for implementing PFK-targeted therapies in clinical practice that incorporates AI-based drug design, nanodelivery, and immune-metabolic modulation.

## 1 Introduction

Warburg effect Cancer cells use aerobic glycolysis to augment glucose uptake and utilization, leading to increased glycolysis (De Leon-Oliva et al., 2025). The Warburg effect, first described by Otto Warburg in 1956, is a key feature of cancer metabolism where cancer cells prefer aerobic glycolysis over oxidative phosphorylation (OXPHOS), even when oxygen is plentiful (Warburg, 1956). This metabolic shift supports rapid cell growth, drives uncontrolled proliferation, and creates an acidic, lactate-rich tumor microenvironment (TME) that helps cancer cells evade the immune system, enhances invasion and metastasis, and leads to resistance to standard therapies (Barba et al., 2024; De Leon-Oliva et al., 2025).

Here, cancer cells in cell culture metabolize high glucose concentrations and secrete lactate under high oxygen levels, unlike normal cells, which maintain the balance between glycolysis and OXPHOS (Hammond et al., 2024). The Warburg effect enhances tumor growth and inhibits the immune response by elevating lactate and lowering pH in the TME, thereby impairing T-cells’ function (Barba et al., 2024). The presence of elevated lactate levels also contributes to metastasis by activating pathways, including nuclear factor kappa B (NF-*κ*B) and phosphoinositide 3-kinase (PI3K)/protein kinase B (AKT) (Gupta P. et al., 2014; Barba et al., 2024). Also, acidosis may induce signals leading to cancer cell invasion, especially via the NF-*κ*B pathway (Gupta S. C. et al., 2014). Several studies have reported that the Warburg effect can control the growth of various cancer types, and that disrupting glucose uptake or aerobic glycolysis can slow the tumor’s development (Li et al., 2025).

PFK catalyzes the conversion of fructose 6-phosphate to fructose 1,6-bisphosphate using Mg-adenosine triphosphate (ATP) as a phosphoryl donor in prokaryotic and eukaryotic cells (Kumar et al., 2026). PFK-1, the key enzyme controlling glycolysis, has three tissue-specific isoforms in mammals: platelet type (PFKP), muscle type (PFKM), and liver type (PFKL). Each isoform shows different expression patterns and plays diverse roles in cancers (Lynch et al., 2024; Yuan et al., 2025). The 6-phosphofructo-2-kinase/fructose-2,6-bisphosphatase (PFKFB) family includes four isoenzymes (PFKFB1–4), with PFKFB3 and PFKFB4 being the most overexpressed in human cancers (Kotowski et al., 2021; Zhang et al., 2025). These bifunctional enzymes regulate the intracellular levels of fructose-2,6-bisphosphate (F2,6BP), which is a highly effective allosteric activator of PFK-1. PFKFB3 has the highest kinase activity, promoting glycolysis, whereas PFKFB4 has stronger phosphatase activity, guiding glucose into the pentose phosphate pathway to maintain redox balance (Zhang et al., 2025).

The PFK-1 and PFKFB families are linked through regulated glycolysis to associate with oncogenic signals, stress pathways, and cancer traits. PFKFB3 and PFKP are upregulated by c-MYC, which links proliferation to glycolysis (Liu et al., 2024; Zhang et al., 2025). ROCK1 stabilizes c-MYC in pancreatic cancer to augment PFKFB3 (Pang et al., 2024). PFKFB3, PFKFB4, and PFKP are regulated by hypoxia-inducible factor (HIF-1*α*) under hypoxic conditions, altering metabolism and leading to tumor resistance (Yi et al., 2019; Kotowski et al., 2021; Zhang L. et al., 2021; Dai T. et al., 2022). The PI3K/AKT/mammalian target of rapamycin (mTOR) pathway promotes glycolysis: AKT phosphorylates PFKFB3 at Ser461 to increase its activity; mTORC1 promotes PFKFB2 to confer cancer stemness (Yi et al., 2019; Fontana et al., 2024; Li et al., 2024). P53 represses PFKFB4, yet p53-deficient cells rely on it to maintain a redox state that becomes vulnerable (Ros et al., 2017). PFKFB3 is phosphorylated by adenosine monophosphate-activated protein kinase (AMPK) under metabolic stress to boost glycolysis and help cells survive mitotic arrest (Doménech et al., 2015). PFKFB3 is approved by the epidermal growth factor receptor (EGFR) pathway and facilitates redox equilibrium, DNA damage repair, and resistance to EGFR inhibitors in non-small cell lung cancer (NSCLC) (Lypova et al., 2024). MiRNAs control the isoforms of PFK: miR-488 silences PFKFB3 and chemoresistance; miR-195-5p silences PFKFB4 (Deng et al., 2021; Sun and Jin, 2022). PFKFB3 is stabilized by ubiquitin-specific peptidase 27 (USP27) in hepatocellular carcinoma (HCC) (Xie et al., 2025); PFKP is degraded by HMG-CoA reductase degradation 1 (HRD1), and its loss enhances breast cancer (BC) aggression (Fan et al., 2021); PFKM is upregulated by Zinc finger E-box binding homeobox-1 (Zhou et al., 2021), which facilitates the Warburg effect and metastasis; PFKM mRNA is altered by N-acetyltransferase-10 and alters glycolysis (Mei et al., 2024). These networks make PFK isoforms critical regulators of metabolism, which present therapeutic targets.

Metabolic targeting in cancer therapy research is also ongoing, aiming to identify small molecules that can suppress key metabolic processes related to cancer growth, especially those in the glycolytic pathway (Pal et al., 2025). This review discusses the functions of the PFK isoforms in cancer development and critically evaluates synthetic and natural PFK inhibitors as therapeutic agents. In addition to listing specific inhibitors, we emphasize that the future of the field lies in capitalizing on the isoform specificity and allosteric regulation of PFK biology, as well as non-metabolic moonlighting functions, to rationally combine and design these compounds into effective anticancer decoys. We also discuss the clinical usefulness of PFK isoforms, current research gaps, and our vision for developing PFK-based precision therapies.

## 2 Purification and characterization of phosphofructokinase

The key roles of PFK in regulating cancer metabolism have prompted numerous studies on its isolation and biochemical research in different species and tissues. Gradually, purification methods have evolved from simpler salt-based fractionation to more complicated affinity-based purification methods. In the early stages, ammonium sulfate precipitation, ion-exchange chromatography, and gel filtration were used (Kuo et al., 1986; Reibstein et al., 1986). The discovery of affinity chromatography was a breakthrough, more so the utilization of immobilized dyes such as Cibacron Blue F3G-A, which binds to the nucleotide-binding site of the enzyme to purify it with high specificity on sources like yeast, rabbit muscle, and *Drosophila melanogaster* (Munneke and Collier, 1985; Kuo et al., 1986; Reibstein et al., 1986). Additionally, PFK was isolated and purified from rats’ white and brown adipose tissues using blue dextran-Sepharose, achieving a 1000-fold purification. The enzymes purified from both tissues showed hyperbolic kinetics with fructose 6-phosphate. They were inhibited by ATP and citrate and activated by adenosine monophosphate (AMP), phosphate, and F2,6BP (Sale and Denton, 1985). Human erythrocyte phosphofructokinase was purified 15,000-fold by ammonium sulfate precipitation, heat treatment, and Sepharose 6-B column chromatography, yielding a preparation with a specific activity of 60 µmol of fructose 1,6-bisphosphate formed per minute per milligram of protein at 25 °C (Staal et al., 1972).

The PFK structural analysis has been important in understanding the role and regulation of this protein. The enzyme is usually found as a tetramer (Kuo et al., 1986; Reibstein et al., 1986). Crystallization experiments using human muscle PFK and other microorganisms have produced detailed atomic structures of active and allosteric regulatory sites (Reibstein et al., 1986). Several tissue-specific isozymes, such as muscle (M), liver (L), and platelet (P), have been described, each with specific kinetic and regulatory properties which have been elucidated by cDNA cloning and recombinant methodology (Vora, 1983).

The kinetic and regulatory investigations emphasize that PFK is an allosteric enzyme that is regulated by various cellular metabolites. AMP, adenosine diphosphate (ADP), inorganic phosphate, and F2,6BP are activators that are critical to cause its active R-state, and F2,6BP is the strongest stabilizer (Reibstein et al., 1986; Thomas and Uyeda, 1986; Lynch et al., 2024). In another study, the human muscle PFK was expressed in a yeast strain lacking PFK genes, purified, buffer-exchanged into a specific solution, and concentrated for crystallization. Crystals were grown in a reservoir containing LiNO_3_, NaF, and sodium acetate at 292 K via vapor diffusion methods, with optimization techniques used to improve quality. Despite success in crystallization, diffraction was limited to 6.0 Å, and crystals showed high solvent content and instability during cryocooling (Kloos et al., 2014).

There are various challenges in purifying PFK because it is highly unstable, prone to aggregation, and likely to be degraded by proteolytic enzymes during extraction, requiring protease inhibitors, substrate analogs, and reducing agents to maintain its functionality (Reibstein et al., 1986). Also, the unique peculiarities of pyrophosphate-dependent PFK isoforms have been exploited to develop sensitive enzyme assays for the detection of F2,6BP (Reibstein et al., 1986; Compton and Patrick, 2025).

Overall, the research and purification of PFK are well established but continue to evolve. The development of affinity purification and structural biology has increased our ability to isolate and study this critical enzyme. Despite ongoing problems with enzyme stability and isoform diversity, modern protocols permit exhaustive biochemical investigation of PFK. This makes it an important area of interest in metabolic research and possible anticancer therapy.

## 3 The phosphofructokinase endogenous regulators

A system of natural activators and inhibitors tightly regulates PFK activity, thereby maintaining metabolic balance (Table 1). Endogenous activators increase glycolytic flux to meet the cell’s energy demands. AMP and ADP send low-energy signals that stimulate PFK, thereby facilitating ATP generation (Lynch et al., 2024; Wang et al., 2024). This activation is also linked with important cellular signaling pathways: the PI3K/AKT pathway responds to insulin by increasing PFKFB3 levels (Riera et al., 2002; He J. et al., 2025), PFKFB3 is activated by estrogen in cancer cells (Sengupta et al., 2018), and PFK activation is the result of epidermal growth factor (EGF) stimulation using the RAS/RAF/MEK/ERK pathway (Dai S. et al., 2022). Additionally, HIF-1*α* increases PFKFB3 transcriptional levels (Montemurro et al., 2019).

**TABLE 1:** Endogenous regulators of phosphofructokinase and their effects.

Categories	Activators/Inhibitors	Key findings	References
Endogenous activators	AMP and ADP	Signal low cellular energy states (high AMP/ADP: ATP ratio) to activate PFK and promote ATP synthesis	Lynch et al. (2024), Wang et al. (2024)
	Hormonal/Growth factor pathways	Insulin: Activates via PI3K/AKT, upregulating FKFB3 Estrogen: Stimulates PFKFB3 in cancers (e.g., breast cancer) EGF: Activates via the RAS/RAF/MEK/ERK pathway	Riera et al. (2002), Sengupta et al. (2018), Dai et al. (2022a), He et al. (2025a)
	Signaling molecules and transcription factors	HIF-1α: Master hypoxia regulator; transcriptionally increases PFKFB3 expression	Montemurro et al. (2019)
Endogenous inhibitors	ATP	High concentrations provide potent feedback inhibition, signaling ample energy availability	Zancan et al. (2008)
	Citrate	Key allosteric inhibitor signaling; abundant biosynthetic precursors (e.g., from the TCA cycle); acts synergistically with ATP.	Usenik and Legiša (2010)
	Acyl-CoA	Modulates PFK activity through both covalent and noncovalent interactions	Jenkins et al. (2011)

Endogenous inhibitors are reduced during periods of high levels of energy or when biosynthetic precursors are abundant. The primary ones are ATP, which acts as an inhibitor on its own at high concentrations (Zancan et al., 2008), and citrate, an allosteric inhibitor that suggests there are sufficient biosynthetic building blocks available, and that ATP collaborates with it (Usenik and Legiša, 2010). Moreover, acyl-CoA also influences PFK activity, doing so in both covalent and non-covalent ways (Jenkins et al., 2011).

PFK is one of the enzymes that are important in the glycolysis process, which affects cellular metabolism and energy production. The regulation of its activity by molecules such as F2,6BP and by allosteric effectors such as ATP and citrate illustrates the complexity of metabolite regulation. Insulin- and EGF-induced signaling pathways also underscore the importance of PFK in normal body processes and diseases such as cancer. PFK is associated with multiple pathological conditions when it is not regulated, which explains its possible use as a treatment option. Besides, it is influenced by exogenous influences, such as hypoxia and inflammatory cues, highlighting the importance of the enzyme in metabolic diseases and cancer and the necessity to investigate its regulation in greater detail.

## 4 A conceptual framework for PFK-targeted cancer therapy

**FIGURE 1:**
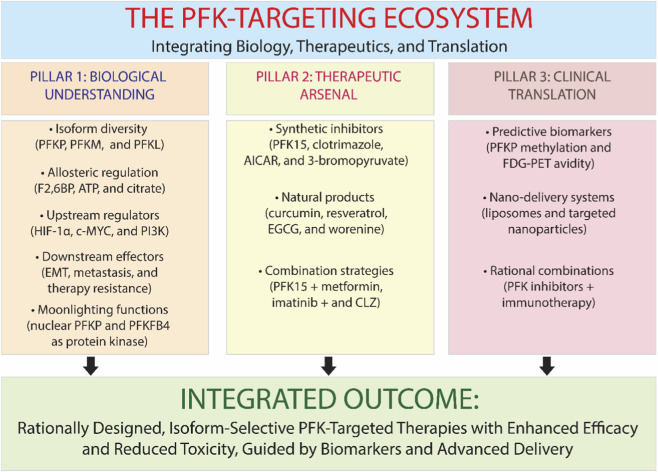
The phosphofructokinase (PFK)-targeting ecosystem. Three pillars of the ecosystem are linked to each other: (1) Biological Understanding - including diversity of PFK isoforms, allosteric regulation, upstream oncogenic drivers, downstream oncogenic processes, and non-metabolic moonlighting activities; (2) Therapeutic Arsenal - such as synthetic small-molecule inhibitors, and natural product-derived compounds, as well as ways to combine them; and (3) Clinical Translation - including predictive biomarkers, advanced nano-delivery systems, and rational combination regimens of these pillars is required to produce next-generation PFK-targeted therapies which are isoform selective, less off-target toxic, and resistant to metabolic plasticity.

To navigate the maze of PFK biology and its therapeutic manipulation, we suggest a conceptual framework, the PFK-Targeting Ecosystem, that sketches the field into three mutually supporting pillars (Figure 1). Pillar 1: Biological Understanding brings together the basic knowledge needed to determine rational therapeutic points of entry. It consists of tissue-specific expression of PFK isoforms (PFKP, PFKM, and PFKL), allosteric regulation of PFK activity by metabolites (F2,6BP), ATP, and citrate, and upstream oncogenic signaling pathways (HIF-1*α*, c-MYC, and PI3K/AKT) that regulate PFK expression and activity (Lynch et al., 2024; Yuan et al., 2025). In addition, the downstream effects of PFK-dependent activation, e.g., epithelial-mesenchymal transition (EMT), metastasis, and therapy resistance, along with the newer recognized moonlighting roles of PFK isoforms in nuclear signaling and protein kinase activities, are also included in this pillar (Gao et al., 2021; Wang H. et al., 2023; Paul et al., 2025).

Pillar 2: Therapeutic Arsenal lists accessible chemical agents for PFK inhibition, classified as synthetic small molecules (e.g., PFK15, clotrimazole, and 3-bromopyruvate) (Coelho et al., 2011; Guo et al., 2016; Deng et al., 2019; Liu et al., 2019) and natural compounds (e.g., curcumin, resveratrol, epigallocatechin-3-gallate (EGCG), and worenine (Li et al., 2016; Li W. et al., 2020; Ghasemi et al., 2019; Ji et al., 2021). The new combination approaches that exploit synthetic lethality or metabolic vulnerabilities, including the synergistic interaction between PFK15 and metformin (Liu et al., 2019) and imatinib and clotrimazole (Motawi et al., 2015). These are also emphasized in this pillar.

Pillar 3: Clinical Translation outlines the key actions required to bring PFK inhibitors to clinical application. These include the development of predictive biomarkers to stratify patients (e.g., PFKP methylation status and 18F-FDG PET), the design of novel nanodelivery systems to enhance bioavailability and tumor targeting, and the development of combination regimens that rationally combine PFK inhibitors with standard-of-care chemotherapy, radiotherapy, or immunotherapy (Xu Y. et al., 2025).

The main assumption of this framework is that the future of PFK-targeted cancer therapy lies not in the individual achievements of each of the three pillars, but in reflecting on the synergy among all three. Strong biological insights (Pillar 1) should inform inhibitor selection and design (Pillar 2), which should be combined with potent translational strategies (Pillar 3) to achieve clinical success. This review follows this ecosystem, and the latter sections align with each pillar and are completed by a discussion of the integrated future directions.

## 5 Phosphofructokinase functions in cancer

PFK enables the proliferation, invasion, and migration of cancerous cells, such as prostate, liver, breast, and colorectal (CRC) cancers, as the cancer cells absorb glucose at an accelerated rate and generate lactate, which increases their growth, invasion, and spread (Ma et al., 2025; Wu et al., 2025; Vincken et al., 2026; Zhang et al., 2026). PFK is a vital component of tumor survival and proliferation across different microenvironments, as it plays a central part in metabolic reprogramming. PFKFB3 and PFKFB4 stimulate aerobic glycolysis by generating F2,6BP to activate PFK allosterically. This enhances glycolytic flux, glucose uptake, and lactate production, thereby sustaining the Warburg effect, a metabolic hallmark of cancer, thus boosting rapid biosynthesis and tumor progression (Yang et al., 2025; Zhang et al., 2025; Sharma and An, 2026).

PFKFB3, PFKP, and PFKFB4 collectively promote tumor progression by enhancing cell proliferation, EMT, migration, and invasion. Importantly, PFKFB3 overexpression is linked to EMT activation and resistance to immunotherapy in CRC (Lu S. et al., 2024). PFKP facilitates metastasis via the AXL-MET receptor tyrosine kinase pathway in NSCLC and induces hypoxia-driven glycolytic reprogramming in BC (Zhao et al., 2024; Anwar et al., 2025). Meanwhile, PFKFB4 acts as a key molecule connecting glycolytic activation to EMT-mediated metastatic spread in pancreatic cancer (Lu et al., 2026). In addition, PFKFB3 and PFKFB4 contribute to therapy resistance by conferring resistance to chemotherapy, radiotherapy, and targeted therapy through increased DNA repair, inhibition of apoptosis (through ferroptosis suppression), and stress-adaptive metabolism (He Z. et al., 2025; Wang J. et al., 2025; Vincken et al., 2026). PFK and PFKFB3 control important stromal components in the TME. It enhances protumor polarization and macrophage immunosuppression, promotes metabolic coupling in tumor-associated macrophages (TAMs) and cancer-associated fibroblasts (CAFs) (Nishi et al., 2025; Shmakova et al., 2025).

In addition to their classical role in glycolysis, the PFK isoforms are also involved in non-metabolic (“moonlighting”) activities that directly affect oncogenic signaling, gene expression, and immune responses. Recent research identified PFKP as a nucleocytoplasmic shuttling protein that contains functional nuclear localization signals and nuclear export sequences (Gao et al., 2021; Liu et al., 2026). Cyclin D3/CDK6-mediated dimerization of the protein facilitates its nuclear entry by exposing the nuclear localization signals and enabling interaction with importin-9 (Liu et al., 2026). PFKP is a transcriptional co-regulator in the nucleus, which increases c-MYC-directed expression of C-X-C chemokine receptor type-4, which facilitates homing and infiltration of leukemia cells into other tissues (Gao et al., 2021; Wang H. et al., 2023). Nuclear PFKP was detected only in invasive T-cell malignancies and not in non-malignant lymphoid tissues; it was also associated with worse patient survival, which suggests it as a diagnostic and therapeutic target (Gao et al., 2021). Equally, PFKFB3 also has functions outside of the production of F2,6BP; it is a signaling scaffold that activates PI3K/AKT/mTOR pathway and Wnt/*β*-catenin pathways, enhancing p-AKT (Ser473), p-GSK3*α*/*β*, and nuclear *β*-catenin, and anaplastic thyroid carcinoma. The effect of the AKT inhibitor MK2206 reverses these effects, indicating that PFKFB3 is upstream of proliferation and migration and independent of, as well as additive to, its glycolytic activity (Deng et al., 2024). PFKFB3 increases programmed death-ligand-1 (PD-L1) expression on peritumoral monocytes via NF-*κ*B activation, thereby promoting immune evasion by inhibiting cytotoxic T cells in the TME, especially in HCC (Chen et al., 2019). PFKFB4 is an intrinsically active kinase with a non-canonical splice variant (PFKFB4-ΔEx6) that directly interacts with AKT at the kinase domain and phosphorylates it, initiating the PI3K/AKT/mTOR signaling pathway without the need to go through glycolysis. This variant is commonly overexpressed in HCC and is associated with poorer prognosis, and increases the sensitivity of tumor cells to mTOR inhibitors, an example of how a metabolic enzyme can develop a kinase function to be used as a therapeutic target (Wan et al., 2025). Altogether, these moonlighting activities outline PFK isoforms as versatile signaling centers that interconnect metabolic state with transcriptional control, signaling, and immune evasion. This implies that future PFK inhibitors must consider both of these non-canonical protein interactions to develop more selective anticancer therapy that can spare normal tissues.

Besides playing an indispensable role in metabolism, certain PFK isoforms also significantly affect resistance to different cancer therapies. For example, trastuzumab resistance in human epidermal growth factor receptor-2 (HER2)-positive BC and cisplatin resistance in gastric cancer have been linked to overexpression of PFKFB3, which inhibits cell death pathways, such as ferroptosis, or promotes DNA repair (He Z. et al., 2025; Vincken et al., 2026). On the same note, PFKFB4 confers resistance to sunitinib in clear-cell renal cell carcinoma and to lenvatinib in HCC, mainly by engaging alternative survival pathways (Feng et al., 2021; Wang J. et al., 2025). This association between PFK activity and resistance to chemotherapy underscores its potential as a target for enhancing tumor response to therapies. Altogether, PFK is a critical metabolic and signaling pathway in cancer (Table 2), and targeting of this protein represents an attractive method to interfere with tumor metabolism and improve therapy.

**TABLE 2:** Roles of phosphofructokinase in cancer progression.

Roles in cancer	Key mechanisms	PFK isoforms	References
Driver of aerobic glycolysis (Warburg effect)	• Produces F2,6BP to activate PFK, the rate-limiting enzyme allosterically. • Increases glycolytic flux, glucose uptake, and lactate production. • Fuels rapid biosynthesis and tumor growth	PFKFB3 and PFKFB4	Zhang et al. (2025), 2026
Promoter of tumor proliferation, invasion, and metastasis	• Enhances cell cycle progression. • Induces Epithelial-Mesenchymal Transition. • Increases cell migration and invasion	PFKP PFKFB3, and PFKFB4	Peng et al. (2023), Deng et al. (2024), Lu et al. (2024a), 2026; Zhao et al. (2024), Anwar et al. (2025), Chen et al. (2025), Lee et al. (2025), Sun et al. (2025)
Contributor to therapy resistance	• Confers resistance to chemotherapy (cisplatin, trastuzumab, and 5-fluorouracil), radiotherapy, and targeted therapies. • Mechanisms: Enhanced DNA repair, inhibition of apoptosis (e.g., via ferroptosis suppression), metabolic adaptation under stress	PFKFB3 and PFKFB4	He et al. (2025b), Wang et al. (2025b), Vincken et al. (2026)
Regulator of the tumor microenvironment	• In macrophages Promotes pro-tumor polarization and immunosuppression (e.g., via PD-L1). • In cancer-associated fibroblasts: Drives metabolic coupling with cancer cells	PFK and PFKFB3	Nishi et al. (2025), Shmakova et al. (2025)
Involvement in non-metabolic (“Moonlighting”) functions	• PFKP: Can localize to the nucleus and regulate gene transcription.• PFKFB3: Influences key signaling pathways (PI3K/AKT and mTOR). • PFKFB4: PFKFB4-ΔEx6 directly phosphorylates AKT1 to facilitate the progression of HCC.	PFKP, PFKFB3, and PFKFB4	Chen et al. (2019), Gao et al. (2021), Deng et al. (2024), Wan et al. (2025), Liu et al. (2026)

The pan-cancer TCGA analysis indicates that the glycolytic enzymes PFKP, PFKM, and PFKL exhibit distinct expression profiles. In PFKP, the expression is highly upregulated in tumor tissues of breast invasive carcinoma (BRCA), cholangiocarcinoma (CHOL), colon adenocarcinoma (COAD), liver hepatocellular carcinoma (LIHC), lung adenocarcinoma (LUAD), stomach adenocarcinoma (STAD), but is downregulated in kidney chromophobe (KICH), and kidney renal clear cell carcinoma (KIRC) (Supplementary Figure 1). PFKM shows elevated expression in cancers such as BLCA, BRCA, and cervical squamous cell carcinoma (CESC), and downregulation in CHOL and COAD samples (Supplementary Figure 2). PFKL is generally upregulated, with the greatest upregulation in CHOL, COAD, esophageal carcinoma (ESCA), and STAD, but the lowest levels in thymoma (THYM) and UCEC, and intermediate levels in BRCA and PAAD (Supplementary Figure 3). This heterogeneity underscores that these major metabolic enzymes are tissue-specific, acting as oncogenic activators in some cancers and suppressors in others, underscoring the critical importance of cancer origin in assessing their potential as therapeutic targets.

## 6 Key genetic and signaling interactions of PFK in cancer

The circuitry of upstream genetic regulators tightly regulates the expression and activity of PFK isoforms, which, in turn, has far-reaching effects on the downstream oncogenic pathways. The key PFK isoforms, PFKFB3, PFKFB4, PFKP, and PFKL, are involved in distinct but overlapping networks of molecular interactions that together drive cancer.

### 6.1 Upstream regulators

**FIGURE 2:**
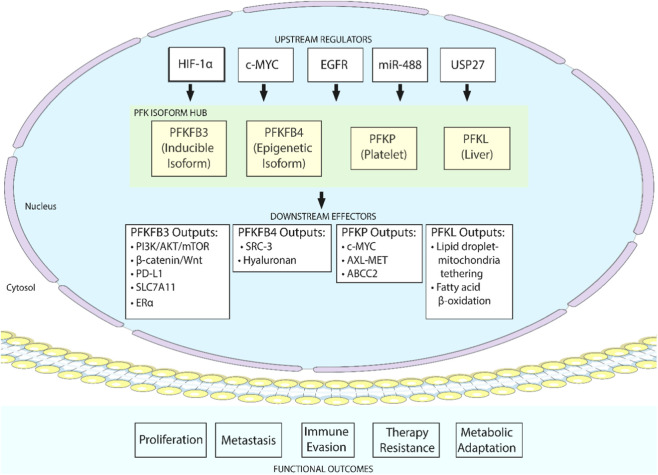
The PFK Regulatory Hub Network. PFK isoforms (PFKFB3, PFKFB4, PFKP, and PFKL) are linked to upstream oncogenic signaling pathways and downstream cancer-associated programs within the PFK regulatory hub network. Hypoxia-inducible factor-1*α* (HIF-1*α*), the master transcriptional regulator c-MYC, receptor tyrosine kinase pathways (such as EGFR), and microRNAs (miR-488 and miR-195-5p) are upstream regulators, as are deubiquitinases (ubiquitin-specific peptidase 27 (USP27)) and protein degradation (HMG-CoA reductase degradation 1 (HRD1)). These regulators focus on individual PFK isoforms, each with its own regulatory landscape. PFKFB3 has isoform-specific downstream effects, including PI3K/AKT/mTOR, Wnt/*β*-catenin, immune evasion via PD-L1, ferroptosis inhibition by SLC7A11/xCT, and endocrine resistance via ER*α*. PFKFB4 enhances metastasis by mediating hyaluronan phosphorylation of steroid receptor coactivator-3 (SRC-3) and hyaluronan-mediated extracellular matrix remodeling. PFKP induces a positive feedback loop with c-MYC, triggers the AXL-MET axis, and increases ATP-binding cassette sub-family C member-2 (ABCC2) expression, thereby driving chemoresistance. PFKL improves the fatty acid *β*-oxidation process through tethering lipid droplets to mitochondria.

PFK isoforms are regulated by important oncogenic post-transcriptional and transcriptional states upstream (Figure 2). Examples include HIF-1*α* (Liu et al., 2025), the master regulator c-MYC (Liu et al., 2024), and receptor tyrosine kinase signaling, including the EGFR, all of which can induce PFKFB3 transcriptionally in NSCLC (Lypova et al., 2024). PFKFB3 is also post-transcriptionally regulated by miR-488, which directly binds the 3′-untranslated region of the PFKFB3 mRNA and decreases PFKFB3 protein levels, thereby inhibiting glycolysis and chemoresistance in CRC cells (Deng et al., 2021). In addition, PFKFB3 expression is stabilized by deubiquitinases such as USP27 (Xie et al., 2025). Likewise, PFKFB4 is transcriptionally regulated by the HIF-1*α* and lysine demethylase-3A/specificity protein 1 (SP1) epigenetic axis (Wang and Wang, 2022), and its mRNA by miR-195-5p (Sun and Jin, 2022). PFKP is regulated by the c-MYC as a transcriptional factor and maintained by the desmosomal protein, plakophilin 1 (PKP1) (Liu et al., 2024; Ritoré-Salazar et al., 2025; Wang et al., 2026), and is degraded by the E3 ligase, HRD1 (Fan et al., 2021). The EMT-transcription factor Zinc finger E-box binding homeobox-1 activates PFKM, while N-acetyltransferase-10 mediates ac4C acetylation and m6A modification via involvement of YTHDC1-LDHA/PFKM, regulating glycolysis (Zhou et al., 2021; Mei et al., 2024). This regulation is a multilayered, dynamically coupled activity of PFK in response to stress signals and the tumor’s metabolic demands.

### 6.2 Downstream targets

PFK isoforms coordinate numerous downstream signaling pathways and oncogenic cascades, driving malignant progression (Figure 2). They may be thematically split into four functional modules:

### 6.3 Basic signal transduction cascades

PFKFB3 serves as a signaling scaffold that activates the PI3K/AKT/mTOR pathway, forming a feed-forward loop that enhances glycolysis and promotes glioblastoma survival (Xia et al., 2025). Meanwhile, PFKFB3 stabilizes *β*-catenin and facilitates its nuclear entry, thus activating the Wnt/*β*-catenin pathway and promoting the growth of anaplastic thyroid carcinoma (Deng et al., 2024). PFKP has a positive feedback loop with c-MYC, in which it increases the stability of c-MYC through the ERK mechanism, and c-MYC upregulates PFKP, resulting in the continued proliferative signaling in head and neck squamous cell carcinoma (Liu et al., 2024).

### 6.4 Immune Evasion

PFKFB3 enhances PD-L1 expression, helping tumor cells evade the immune system. PFKFB3 is overexpressed in CRC, which is a predictor of immunotherapy resistance and an immunosuppressive TME (Lu S. et al., 2024). Moreover, ferroptosis may be prevented by PFKFB3, which can dephosphorylate SLC7A11/xCT at serine 26, thereby sustaining cystine uptake and glutathione production, to cause cisplatin resistance in gastric cancer (He Z. et al., 2025). Besides, PFKFB3 stabilizes estrogen receptor-alpha in ER-positive breast tumors, leading to endocrine resistance in conditions of estrogen deprivation (Jia et al., 2024).

### 6.5 Differentiation, metastasis, and cellular stress adaptation

**FIGURE 3:**
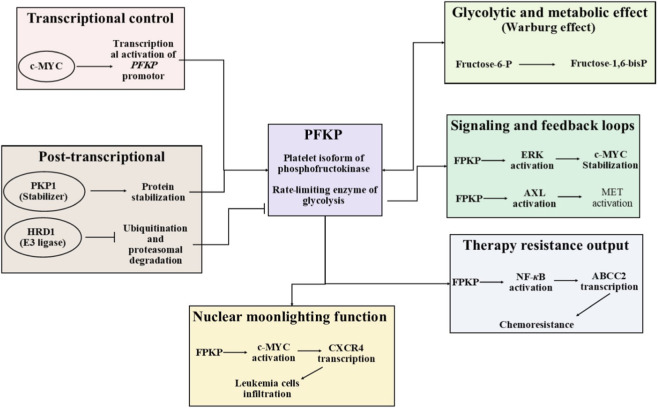
Mechanistic diagram of PFKP-driven oncogenic signaling and metabolic changes. PFKP is transcriptionally enhanced by c-MYC and stabilized after translation by plakophilin 1 (PKP1), while HMG-CoA reductase degradation 1 (HRD1) tags it for degradation. It promotes aerobic glycolysis (Warburg effect), supporting biosynthesis and ATP production. Additionally, PFKP activates the AXL-MET axis to facilitate invasion, establishes a positive feedback loop with c-MYC via ERK signaling, increases ATP-binding cassette sub-family C member-2 (ABCC2) expression via NF-*κ*B to confer chemoresistance, and translocates to the nucleus to co-activate C-X-C chemokine receptor type-4 (CXCR4) transcription, aiding tumor cell homing. These functions link together, positioning PFKP as a key regulator of tumor development and therapy resistance.

PFKFB4 possesses protein kinase activity, and its phosphorylation of steroid receptor coactivator-3 at serine 857 increases its transcriptional activity and gene expression, thereby promoting BC metastasis (Dasgupta et al., 2018). Moreover, PFKFB4 enhances hyaluronan synthesis, which facilitates rearrangement of the extracellular matrix and facilitates invasion (Gao et al., 2018). PFKP also interacts with AXL, activating AXL-MET receptor tyrosine kinase signaling by binding to AXL and facilitating its phosphorylation at Y779, which causes invasive phenotypes in NSCLC (Zhao et al., 2024), as illustrated in Figure 3. PFKL moonlights as a kinase, phosphorylating perilipin-2 on lipid droplets under nutrient-deprived conditions, which promotes lipid droplet-mitochondria tethering. This mechanism facilitates fatty acid mobilization and *β*-oxidation, another energy source to keep tumor cells alive and growing in HCC (Meng et al., 2024).

### 6.6 Therapy resistance mechanisms

PFKP directly promotes ATP-binding cassette sub-family C member-2 expression by stimulating NF-*κ*B, which plays a role in cisplatin resistance in NSCLC (Wang Z. et al., 2023). PFKFB3 resistance to HER2-targeted therapy is associated with trastuzumab resistance in BC, and this resistance is maintained by PFKFB3 (Vincken et al., 2026). In the meantime, metabolic reprogramming mediated by PFKFB4 was found to confer resistance to sunitinib in clear-cell renal cell carcinoma and to lenvatinib in HCC (Feng et al., 2021; Wang J. et al., 2025). This creates a complex network of signal transduction, immune modulation, metabolic adaptation, and drug resistance, making PFK isoforms a therapeutic target.

This two-way interactome indicates the importance of PFK isoforms as central regulatory hubs that receive upstream oncogenic inputs, including HIF-1*α*, c-MYC, and PI3K/AKT, and convert them into the coordinated activation of the numerous downstream pathways. These pathways involve metabolic reprogramming, proliferation signaling, EMT, immune evasion, and therapy resistance, with PFK at the center of cancer cell metabolism and malignant behavior. This network demonstrates that blocking PFK, particularly PFKFB3 and PFKFB4, prevents not only glycolysis but also a variety of cancer-promoting functions. Thus, the use of these isoform inhibitors in combination with the current standard therapies is a promising translational approach. For example, the PFK15 inhibitor PFKFB3 synergizes with metformin in the treatment of myeloma and with oxaliplatin in the production of CRC cells. Also, anti-PFKFB3 therapy can enhance anti-PD-1/PD-L1 in immunotherapy. PFKFB3 inhibitors are useful for treating metabolic adaptation but can be combined with glutaminase inhibitors or OXPHOS blockers, whereas use in combination with radiotherapy, cisplatin, or trastuzumab can help overcome therapy resistance. The combination therapies with these supported modes help the clinical development of PFK-targeted therapies.

## 7 Phosphofructokinase in clinical studies on cancer

Advancing PFK-targeted therapies toward clinical application (Pillar 3) requires evidence of their utility in patient settings. Here, we review clinical studies linking PFK isoforms to diagnosis and treatment response. PFK isoforms have been shown to be clinically relevant across a variety of cancer settings, both in altering treatment response and as a diagnostic marker. PFKFB3 is another promising field of research for improving the results of radiotherapy. A 2025 analysis of the randomized SweBCG91RT BC trial showed that tumors with high nuclear PFKFB3 protein and mRNA levels had the most significant decrease in ipsilateral breast tumor recurrence after adjuvant radiotherapy. This association suggests a link between PFKFB3 expression and radiosensitivity, but does not prove causality. The hypothesis that PFKFB3 influences radiotherapy response, possibly via DNA repair, needs validation through functional studies. However, research has found that PFKFB3 cannot be used independently as a predictive biomarker of the benefits of radiotherapy, because no statistically significant interaction was observed. The expression, however, was more prevalent in HER2-positive and luminal A tumors (Egelberg et al., 2025).

Another PFK isoform, PFKP, has also been confirmed in a non-invasive blood-based test of HCC. In 2019, a Phase II clinical validation demonstrated that a methylated DNA marker, a six-member panel including PFKP, has the potential to diagnose HCC in plasma samples with high accuracy. This panel achieved an AUC of 0.96 on the receiver operating characteristic (ROC) curve, indicating excellent diagnostic accuracy. This significantly exceeds the performance of the standard serum alpha-fetoprotein test, which has an AUC of about 0.80 in large meta-analyses and shows high sensitivity at all cancer stages, from very early to advanced. This indicates that methylation of PFKP, among other markers, is a key component of a liquid biopsy technique for detecting early HCC (Kisiel et al., 2019).

All these studies point to the clinical importance of PFK family members in cancer. PFKFB3 is also a biomarker under investigation as a therapeutic agent to improve radiotherapy in BC. Simultaneously, PFKP methylation is a diagnostic marker in a multi-analyte panel to detect liver cancer. This emphasizes the broader applications of the components of the glycolytic pathway in cancer biology, as well as their increased potential for therapy customization and early cancer detection.

## 8 Phosphofructokinase inhibitors for cancer therapy

**FIGURE 4:**
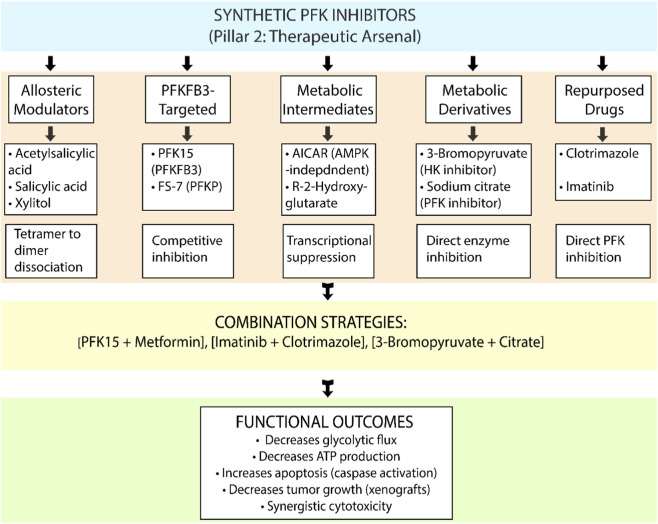
The Synthetic PFK Inhibitor Landscape. A mechanistic taxonomy of synthetic PFK inhibitors organized by their mode of action.

Pillar 2 of our framework encompasses the therapeutic arsenal available for PFK inhibition. We begin with synthetic small-molecule inhibitors, including synthetic intermediates, metabolic intermediates, and sugar substitutes, as shown in Table 3; Figure 4.

**TABLE 3:** Synthetic inhibitors for phosphofructokinase (PFK) in different types of cancer.

Compounds	Key findings on PFK inhibition and anticancer effects	Limitations	Future recommendations
Acetylsalicylic acid and salicylic acid	Promote dissociation of PFK active tetramers into inactive dimers. Decrease glucose consumption and inhibit PFK activity in MCF-7 BC cells (Spitz et al., 2009) FS-7 (a related compound) downregulates PFKP protein in gastric carcinoma MGC-803 cells (Deng et al., 2019)	Lack of specificity; broad anti-inflammatory roles lead to off-target effects	Drug repurposing informed by mechanistic insights; test in trials targeting patients with glycolytic tumor signatures
AICAR	Enhances cytotoxicity of PFK-15 in colorectal cancer cells (SW480, HT29) (Yan et al., 2022)	The mechanism is AMPK-independent and context-dependent, highlighting the complexity of signaling networks	Utilize omics profiling to identify predictive biomarkers for patient selection
Cyclic polylactate	Decreases PFK and HK activity in leukemic cells. Increase caspase activity (Harada et al., 2005)	Most evidence from *in vitro* studies does not recapitulate the complex TME.	Prioritize translational studies using patient-derived xenografts (PDXs) to connect lab findings to clinical applications
Clotrimazole (alone or with Imatinib)	Inhibits PFK in human BC tissue samples (Coelho et al., 2011) A synergistic combination with Imatinib decreases PFK-1 gene expression in T47D BC cells (Motawi et al., 2015)	Lack of specificity; broad antifungal roles lead to off-target effects	Rational combination therapies, e.g., with imatinib, explore immunotherapy opportunities by reducing lactate-driven immunosuppression
R-2-hydroxyglutarate	Suppresses glycolysis and downregulates PFKP expression in acute myeloid leukemia cells (Qing et al., 2021)	Translational gap from *in vitro* to *in vivo* models; compensatory metabolic pathways are not fully addressed	Use omics profiling to identify biomarkers, such as elevated PFKP/FTO expression, for personalized patient selection
PFK15	Competitive inhibitor of PFKFB3. Inhibits glycolysis, kills multiple myeloma and lung adenocarcinoma cells, and reduces tumor growth in xenograft models (Zhu et al., 2016; Li et al., 2018; Liu et al., 2019)	Pharmacokinetic challenges, e.g., the need for intraperitoneal injections in mouse models, may trigger compensatory pathways, such as glutaminolysis	Design next-generation inhibitors targeting PFKFB3; prioritize combination therapies (e.g., with metformin, conduct detailed pharmacokinetic and PDX studies for clinical translation
Sodium citrate (with 3-bromopyruvate)	Sodium citrate inhibits PFK activity. Combined with 3-bromopyruvate, it induces cytotoxicity in gastric cancer MGC-803 cells (Guo et al., 2016)	The evidence is limited to *in vitro* models (e.g., MGC-803 cells) and does not recapitulate the TME.	Systematic investigation of PFK isoforms using genetic tools for isoform-specific drug development
Xylitol	Suppresses oral squamous cell carcinoma proliferation. In low glucose, PFK activity decreases, leading to apoptosis (Trachootham et al., 2017)	Cancer cell metabolic plasticity may allow therapeutic escape via compensatory pathways	Explore rational combination therapies to overcome metabolic plasticity; investigate immunotherapy synergy by modulating lactate levels

### 8.1 Acetylsalicylic acid and salicylic acid

Acetylsalicylic acid (ASA; aspirin) and its active metabolite, salicylic acid, are NSAIDs with antitumor effects. In addition to their typical COX-mediated mechanisms, both drugs also target the glycolytic pathway, as initially mapped by Spitz et al. (2009)—showing that ASA and SA inhibit PFK in a dose-dependent manner. They promote the conversion of active tetrameric PFK into inactive dimers, directly reducing the Warburg effect. This structural shift decreases glucose uptake and cell growth in human BC (MCF-7) cells. Building on this, Li et al. (2017) found that aspirin can reverse sorafenib resistance in HCC by inhibiting PFKFB3, a key allosteric activator of PFK. This results in lowered glycolytic flux and enhanced drug sensitivity both *in vitro* and *in vivo*. These findings show that ASA and SA inhibit the Warburg effect through a dual mechanism: direct allosteric modulation of PFK-1’s structure and indirect inhibition of PFKFB3. Also, Deng et al. (2019) examined the anticancer activity of FS-7, a flavonoid salicylate derivative, in the MGC-803 human gastric carcinoma cell line. The authors indicated that FS-7 inhibited MGC-803 colony growth and cell motility and reduced the protein expressions of HIF-1*α*, hexokinase (HK)-II, and PFKP.

### 8.2 AICAR

AICAR (5-amino-4-imidazole carboxamide riboside) is known to cause apoptosis in various types of tumor cells. AICAR stimulated the cytotoxicity of the human CRC cells (SW480 and HT29) induced by PFK-15 (1-(4-pyridinyl)-3-(2-quinolinyl)-2-propen-1-one) through an AKT-dependent mechanism, while this effect was independent of AMPK (Yan et al., 2022).

### 8.3 Cyclic polylactate

Cyclic polylactate is a supramolecular oligomer identified in the culture medium of HeLa tumor cells and reported to inhibit the glycolytic pathway. Cyclic polylactate reduced the activities of PFK and HK in leukemic cells. In addition, it increases the activities of caspases 3, 8, and 9 (Harada et al., 2005).

### 8.4 Imatinib mesylate and clotrimazole

Clotrimazole is an antifungal azole derivative that antagonizes the glycolytic enzymes during the treatment of cancer. A synergistic effect was observed when imatinib mesylate and clotrimazole were combined in human BC (T47D) cells. This interaction resulted in inhibition of glycolysis, as evidenced by decreased gene expression and ATP levels in T47D cells treated with the combination compared with the untreated cells (Motawi et al., 2015). Furthermore, Coelho et al. conducted a study using only clotrimazole on human BC tissue samples. They observed that PFK activity was suppressed by clotrimazole (Coelho et al., 2011).

### 8.5 R-2-hydroxyglutarate

R-2-hydroxyglutarate is a product of the mutation of isocitrate dehydrogenases in anticancer activity. Findings indicated that R-2-hydroxyglutarate is a promising treatment for leukemia. R-2-hydroxyglutarate inhibited glycolysis and silenced fat mass- and obesity-related gene (FTO)/PFKP/lactate dehydrogenase (LDH)-B expression in human primary isocitrate dehydrogenases-wild-type acute myeloid leukemia (Qing et al., 2021).

### 8.6 PFK15

PFK15 (1-(4-Pyridinyl)-3-(2-quinolinyl)-2-propen-1-one) is a competitive inhibitor of PFKFB3, the major enzyme for F2,6BP production, leading to decreased glycolysis. *In vitro*, PFK15 inhibited human multiple myeloma cell lines (RPMI8226, ARP-1, and OPM2) through the PFKFB3/MAPK/STAT axes and enhanced the anti-myeloma effect of metformin (Liu et al., 2019). Furthermore, PFK15 reduced the rate of glycolysis in human lung adenocarcinoma A549 cells, reflected by reduced glucose uptake, F2,6BP, and lactate levels (Li et al., 2018). *In vivo*, intraperitoneal administration of PFK15 (25 mg/kg) decreased tumor weight and volume in a gastric cancer xenograft model by a dose of 25 mg/kg (Zhu et al., 2016). Thus, PFKFB3 inhibition is a promising avenue.

### 8.7 Sodium citrate and 3-Bromopyruvate

Sodium citrate suppressed the growth of human gastric adenocarcinoma epithelial cells by suppressing glycolysis (Xia et al., 2018). The 3-bromopyruvate-sodium citrate mixture induced cytotoxicity, ATP production, and lactate production in the human gastric cancer cell line, MGC-803. In addition, 3-bromopyruvate, on its own, enhanced inhibition of HK activity, whilst sodium citrate inhibited PFK activity (Guo et al., 2016).

### 8.8 Xylitol

Xylitol, a pentose sugar alcohol, is used as a sugar and glucose substitute and can specifically suppress the growth of oral squamous cell carcinoma cells while leaving normal oral keratinocytes untouched. While it has equal energy content, cancer cells grown on a low glucose medium with xylitol have lower ATP levels, PFK activity, and glycolytic flux, suggesting xylitol is not effectively used in glycolysis to promote cancer growth (Trachootham et al., 2017). In a mouse model of oral cancer, substituting glucose with xylitol at a typical dose for humans (around 10 g per day) increased median survival from 19 to 30.5 days and reduced Ki-67 and PFK-1 expression, suggesting it could be used as a sweetener for oral cancer survivors (Sahasakul et al., 2022). However, cancer cells can circumvent glycolytic inhibition through other metabolic pathways such as OXPHOS and glutaminolysis, so it is unclear whether chronic use of xylitol as the only treatment for cancer is safe and effective in animals and patients.

## 9 Natural products as PFK inhibitors for cancer therapy

**FIGURE 5:**
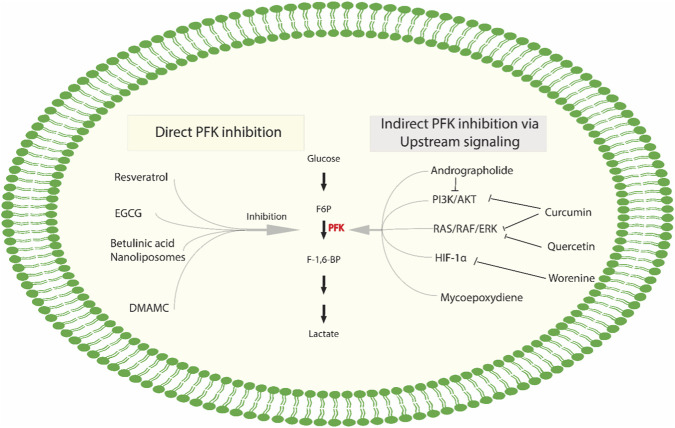
Natural product inhibitors targeting phosphofructokinase (PFK) in cancer.

Complementing synthetic agents, natural product-derived compounds constitute a second major class within the therapeutic arsenal (Pillar 2). Natural products have been widely used in cancer treatment and target several signaling mechanisms. We shall discuss the application of natural products as PFK inhibitors in cancer treatment (Table 4; Figure 5).

**TABLE 4:** Natural product inhibitors for phosphofructokinase (PFK) in different types of cancer.

Compounds	Key findings on PFK inhibition and anticancer effects	Limitations	Future recommendations
Andrographolide	Induces mitochondrial apoptosis and inhibits PFK in lung adenocarcinoma cells (H1975). Acts via multiple pathways (e.g., PI3K/AKT) that can affect PFK (Chen et al., 2021)	Lack of specificity; pleiotropic activity affects multiple pathways; poor pharmacokinetics (bioavailability, rapid metabolism)	Direct enzymatic assays and structural studies to confirm PFK binding; pharmacokinetic optimization via nano-delivery systems
Betulinic acid	Betulinic acid suppresses glycolysis indirectly via NF-κB, PI3K/AKT, and c-MYC pathways (not by directly targeting PFK), while also modulating transcription factors and enhancing chemo-efficacy in colon cancer (Jung et al., 2007; Chintharlapalli et al., 2011; Potze et al., 2016; Jiao et al., 2019; Zheng et al., 2019; Banerjee et al., 2024)	Limited evidence for direct PFK binding; isoform specificity not explored	Investigate isoform selectivity; extend to *in vivo* models; explore combination therapies
Curcumin	Downregulates glycolysis proteins (Mittal et al., 2020) Targets signaling pathways (Ras, PI3K/AKT) that activate PFK, suggesting direct or indirect PFK inhibition (Ghasemi et al., 2019)	Pleiotropic effects; poor bioavailability; rapid metabolism; lack of direct PFK binding evidence	Use CETSA or direct-binding assays; improve delivery via nanoformulations; conduct metabolic phenotyping (e.g., stable isotope-resolved metabolomics)
Dimethylaminomicheliolide	Suppresses aerobic glycolysis and targets PFKL in neuroblastoma (Zhang et al., 2021b)	Limited data on specificity and pharmacokinetics; isoform selectivity unclear	Validate PFKL engagement; assess isoform-specific effects; explore *in vivo* efficacy and combination strategies
Epigallocatechin-3-gallate (EGCG)	Inhibits PFK activity and mRNA levels in breast cancer cells. Modulates PFK oligomeric structure, inducing apoptosis in hepatocellular carcinoma (Li et al., 2016)	Pleiotropic activity; unclear direct PFK inhibition; pharmacokinetic challenges	Perform direct enzymatic assays; investigate isoform specificity; develop advanced delivery systems for better bioavailability
Mycoepoxydiene	Induces apoptosis in HeLa cells by downregulating PFKM (Jin et al., 2017)	Limited mechanistic insight: unknown if effects are direct or indirect; no isoform specificity data	Confirm PFKM targeting via structural studies; evaluate isoform selectivity; test *in vivo* models
Quercetin	Inhibits Ras/Raf and PI3K/AKT pathways (Dhanaraj et al., 2021)	Pleiotropic effects; lack of direct PFK binding evidence; pharmacokinetic limitations	Direct binding validation; metabolic phenotyping to link PFK inhibition to glycolytic flux; explore combination with chemotherapy
Resveratrol	Directly inhibits PFK activity in MCF-7 breast cancer cells, disrupting glucose metabolism. Also inhibits the PI3K/AKT pathway (Gomez et al., 2013; Wang et al., 2020)	Poor bioavailability, rapid metabolism, and pleiotropic effects may confound the interpretation of PFK-specific effects	Optimize pharmacokinetics via nano-delivery; validate PFK inhibition *in vivo*; study isoform-specific effects
Worenine	Reduces the activity and expression of PFK-L in colorectal cancer cells, inhibiting glycolysis and proliferation by targeting HIF-1*α* (Ji et al., 2021)	Limited evidence for direct PFK binding; isoform specificity not fully explored; lacking *in vivo* validation	Confirm direct PFK-L engagement; investigate isoform selectivity; conduct *in vivo* studies and assess combination potential

### 9.1 Andrographolide

Andrographolide is a diterpenoid extracted from the plant *Andrographis paniculata*, which has been shown to possess anti-inflammatory, antiviral, and anticancer activities. Andrographolide was shown to induce mitochondrial apoptosis and downregulate glycolysis in human lung adenocarcinoma H1975 cells; interestingly, PFK gene expression was increased under these conditions, as measured by qRT-PCR, suggesting that the decrease in glycolytic activity is not a result of direct inhibition of PFK gene expression or activity (Chen et al., 2021). Instead, the evidence is mounting that andrographolide indirectly inhibits glycolysis via the PI3K/AKT/mTOR pathway, which is a key positive regulator of glycolytic enzyme expression and activity. In particular, andrographolide suppresses glycolysis and increases the radiosensitivity of CRC cells by inhibiting the PI3K/AKT/mTOR pathway (Li X. et al., 2020). A recent, comprehensive review also confirms that andrographolide inhibits cancer metabolism through the PI3K/AKT/mTOR and HIF-1 pathways (Shaharudin et al., 2024). Finally, andrographolide also downregulates PFKFB3, the most potent allosteric activator of PFK-1, in endothelial cells, and this effect is enhanced in the presence of the PFKFB3 inhibitor, 3-PO, indicating that andrographolide acts upstream of PFK rather than inhibiting it directly (Yao et al., 2019). Therefore, the suppression of PFK activity and glycolytic flux in andrographolide-treated cancer cells is a secondary effect of the inhibition of PI3K/AKT/mTOR pathway and PFKFB3, rather than the direct action of andrographolide on the PFK enzyme itself.

### 9.2 Betulinic acid

Betulinic acid is a pentacyclic lupane-type triterpenoid with well-known anti-cancer properties, primarily via induction of mitochondrial-mediated apoptosis (Gao et al., 2025). As for suppressing glycolysis, it has been reported that betulinic acid does not directly interact with PFK; rather, PFK downregulation is a consequence of its effects on upstream signals. In BC, betulinic acid downregulated aerobic glycolysis by increasing caveolin-1 expression, thereby blocking the NF-*κ*B/c-MYC pathway and downregulating glycolytic proteins, including c-MYC, LDH-A, and PDK1 (Jiao et al., 2019). Similarly, betulinic acid was shown to activate glucose-regulated protein-78, leading to endoplasmic reticulum stress, which induces eIF2*α* phosphorylation, *β*-catenin inactivation, and a reduction in c-MYC-mediated glycolysis and metastasis in BC cells (Zheng et al., 2019). Hence, PFK-1 could be considered part of a multiple-glycolytic-enzyme downregulation program rather than a primary target of betulinic acid. In line with this, mechanistic reviews demonstrate that betulinic acid primarily regulates glycolytic pathways via NF-*κ*B, PI3K/AKT, and c-MYC-dependent transcription pathways, which together downregulate several glycolytic enzymes, making the effect on the PFK enzyme secondary (Banerjee et al., 2024). Further, betulinic acid downregulated stearoyl-CoA desaturase in colon cancer stem cells (Potze et al., 2016), and via downregulation of SP1, SP3, and SP4 transcription factors in colon cancer (RKO and SW480) cells (Chintharlapalli et al., 2011), and enhances the efficacy of 5-fluorouracil and irinotecan in chemoresistant colon cancer cells (Jung et al., 2007).

### 9.3 Curcumin

Curcumin is a bioactive polyphenolic compound extracted from the rhizome of *Curcuma longa* L. (turmeric), a perennial herb belonging to the Zingiberaceae family. It demonstrates various biological activities, including anticancer, anti-inflammatory, and antioxidant effects (El-Saadony et al., 2025). Curcumin and electrical pulses induced apoptosis and reduced clonogenic capacity of triple-negative BC (MDA-MB-231) cells. In addition, the proteomic analysis showed that the glycolysis proteins were downregulated (Mittal et al., 2020). Curcumin has been found to indirectly inhibit glycolysis via upstream pathways. In hepatic stellate cells, curcumin suppresses HK and PFK2 expression via AMPK activation, thereby reducing glycolytic flux (Lian et al., 2016). In cancer cells, curcumin inhibits the Ras, PI3K/AKT, and Wnt/*β*-catenin pathways (Ghasemi et al., 2019), known upstream regulators of PFK expression and activity (Ward and Thompson, 2012). Further, curcumin decreases NF-*κ*B activity, thereby indirectly downregulating the expression of glycolytic enzymes (Ghasemi et al., 2019). In this regard, curcumin likely exerts its anti-glycolytic and apoptotic effects mainly by indirectly targeting PFK.

### 9.4 Dimethylaminomicheliolide

Dimethylaminomicheliolide (DMAMC) is a semi-synthetic guaianolide sesquiterpene lactone derived from micheliolide, a natural product isolated from *Tanacetum parthenium*; a prodrug of the anti-inflammatory sesquiterpene lactone micheliolide (Li et al., 2022). DMAMC demonstrated anticancer activity *in vitro* and *in vivo* on neuroblastoma by inhibiting aerobic glycolysis and attacking PFKL (Zhang S. et al., 2021). DMAMC diminished viability, cell cycle arrest, and cell invasion, as well as EMT of HCC cells, by various procedures (Yao et al., 2020). In addition, DMAMC could be mediated by Bim, the NF-*κ*B pathway, and reactive oxygen species in rhabdomyosarcoma in combination with vincristine or epirubicin (Xu et al., 2019).

### 9.5 Epigallocatechin-3-gallate

EGCG is a bioactive polyphenolic catechin found in green tea (*Camellia sinensis*) extract, known for its various anticancer effects. It markedly reduces the activity and expression of enzymes, such as HK, PFK, and LDH, with a lesser impact on pyruvate kinase, in BC 4T1 cells. Moreover, EGCG directly modifies PFK’s oligomeric structure by encouraging the disassembly of active tetramers into inactive dimers, as shown by size-exclusion chromatography. This process leads to metabolic stress and promotes apoptosis in HCC cells (Li et al., 2016). In addition, EGCG inhibited Ras in human thyroid carcinoma (Wu et al., 2019), and the AMPK in human lung cancer cells (Chen et al., 2020) and c-MYC in leukemia (Della Via et al., 2021), while peracetylated-EGCG targeted the PI3K/AKT in human skin cancer cells (Chiou et al., 2013).

### 9.6 Mycoepoxydiene

Mycoepoxydiene is an isolated natural compound extracted from a marine fungus and has shown good *in vitro* inhibitory effects on HeLa cells. According to Jin et al. (2017), mycoepoxydiene induced dose-dependent apoptosis in cervical cancer (HeLa) cells by downregulating HK2, PFKM, and LDHA. In addition, mycoepoxydiene suppressed AKT in HeLa (Lin et al., 2015).

### 9.7 Quercetin

Quercetin is a naturally occurring flavonol, a type of flavonoid found widely in fruits, vegetables, and grains. It has notable antioxidants, anti-inflammatory, and anticancer properties (Cho et al., 2025). Quercetin treatment of MDA-MB-231, a triple-negative BC cell, inhibited the PFKP–LDHA signaling pathway, thereby reducing the increase in cell migration driven by aerobic glycolysis (Umar et al., 2020). This supports a wider body of research indicating that the PFK-dependent Warburg effect plays an active role in enhancing tumor cell motility and invasiveness (Anwar et al., 2025). In addition, the proliferation and cytotoxic effects of quercetin (50 mM and 75 mM) on human metastatic ovarian cancer (PA-1) cells were inhibited by targeting PI3K/AKT, Ras/Raf, and EGFR expression (Dhanaraj et al., 2021). Also, quercetin inhibited the metastasis of epidermoid carcinoma (A431-III) cells by blocking the AKT/mTOR/c-MYC pathway (Chen et al., 2018). PFK and cancer cell metabolism could be regulated using quercetin to target Ras, c-MYC, and PI3K/AKT.

### 9.8 Resveratrol

Resveratrol is a non-flavonoid polyphenolic stilbenoid that naturally occurs as a phytoalexin in grapes, berries, peanuts, and other plants. It exhibits a wide range of biological activities, including anticancer, anti-inflammatory, and cardioprotective effects (Ren et al., 2025). Gomez et al. (2013) demonstrated that resveratrol directly inhibits PFK enzymatic activity in the MCF-7 human BC cell line, disrupting glucose metabolism, lowering ATP levels, and decreasing cell viability. Resveratrol also increased the sensitivity of CRC (HCT116 and CT26) cells to cetuximab by suppressing PI3K/AKT (Wang et al., 2020). On the same note, resveratrol suppressed cell viability and induced apoptosis of small-cell lung cancer (H446). It suppressed PI3K/AKT/c-MYC expression, suggesting a promising application of resveratrol in the treatment of lung cancer (Li W. et al., 2020).

### 9.9 Worenine

Worenine is an isoquinoline alkaloid derived from the dried rhizome of *Coptis chinensis* that was reported to exhibit anticancer effects. It reduced glucose intake, lactate generation, and the activities of PFK-L, HK2, and PKM2 in CRC (HCT116 and SW620) cells. Moreover, the worenine was used to target HIF-1*α* and prevent the growth of CRC cells (Ji et al., 2021).

## 10 Challenges and research gaps of phosphofructokinase targeting with future recommendations for cancer therapy

Having established the biological basis (Pillar 1), therapeutic arsenal (Pillar 2), and early clinical evidence (Pillar 3), we now address the barriers to integration across this ecosystem and propose strategies to overcome them (Table 5). The primary challenge is achieving isoform specificity, as several PFK isoforms play tissue-specific roles. Normal tissues could be damaged by non-selective inhibition. Previous reviews by Sharma and An (2026) and Ishaq et al. (2025) have highlighted the need for isoform-specific targeting. Our review provides a consolidated analysis of the distinct oncogenic roles of PFKP, PFKM, and PFKL across different cancer types (as shown in our TCGA analysis) and evaluates the potential of emerging compounds like DMAMC and worenine to achieve this selectivity. To address this, future research must use AI-assisted drug design to generate inhibitors and perform single-cell proteomics in conjunction with CRISPR screening to comprehensively identify the relevance of each isoform within different cell types and in cancer cell environments (Zhang et al., 2024; Das, 2025; Menon et al., 2025).

**TABLE 5:** Research gaps and future recommendations for the proper targeting of phosphofructokinase.

Challenges/Research gaps	Description	Future recommendations	References
Isoform-specific selectivity	Multiple PFK isoforms with tissue-specific roles; non-selective inhibition causes toxicity	Use AI-driven drug design to develop isoform-specific inhibitors. Employ single-cell proteomics and CRISPR screens to map isoform essentiality across cell types	Zhang et al., 2024; Das (2025), Menon et al. (2025)
Metabolic plasticity and resistance	Cancer cells switch to alternative pathways (e.g., OXPHOS and glutaminolysis) upon inhibition of glycolysis	CRISPR-based synthetic lethality screens to identify compensatory pathways. Spatial metabolomics to map metabolic rewiring in real time	Cadassou and Jordheim (2023), Wang et al. (2025a), Karpe et al. (2026)
Tumor microenvironment influences	PFKFB3/4 in stromal cells promotes immune evasion and therapy resistance	Spatial transcriptomics and multiplex imaging to dissect PFK expression in stromal vs. tumor cells. Organoid/co-culture models to test stromal-targeting combos	Vu et al., 2022; Faial (2024), Du and Yang (2025)
Therapeutic delivery and specificity	Off-target effects due to poor tumor specificity of inhibitors	Nanoparticle delivery systems with PFK-specific ligands. PFK-targeted PET tracers for imaging and theranostic applications	Ogura et al. (2015), Xu et al. (2025b)
Biomarker deficiency	Lack of predictive biomarkers for patient stratification	Multi-omics integration (scRNA-seq, proteomics, and metabolomics) to identify response signatures. Machine learning models to predict PFK dependency	Lu et al. (2024b), Shi et al. (2025)
Combination therapy optimization	Unclear how to best combine PFK inhibitors with chemo/radio/immunotherapy	High-throughput drug screening in patient-derived organoids. Digital twin models to simulate combination effects *in silico*	Hernandez-Boussard et al. (2021), Stahlberg et al. (2022)
Non-metabolic functions of PFK isoforms	PFK isoforms have roles in transcription, the cell cycle, and DNA repair	Phosphoproteomics and post-translational modification mapping to uncover non-canonical signaling networks. CRISPR-based functional genomics to link isoform functions to phenotypes	Shang et al. (2025)
Resistance via compensatory pathways	Upregulation of other glycolytic enzymes or survival pathways (e.g., PI3K/AKT)	Polypharmacology approaches with multi-omics validation. Live-cell biosensors to monitor metabolic flux dynamically after treatment	Lu et al. (2024b), 2026
Limited clinical translation	Few PFK inhibitors are in clinical trials; human efficacy is unclear	Accelerate trials with biomarker-enriched cohorts. Use of PFK-specific imaging for pharmacodynamic monitoring in trials	Ogura et al. (2015)
Heterogeneity across cancer types	PFK dependency varies widely across and within cancer types	Pan-cancer dependency maps using CRISPR and transcriptomic data. AI-based classification of PFK-driven subtypes	Shi et al. (2025), Xu et al. (2025a)
Impact on immune cells	PFKFB3 inhibition may impair T-cell function and antitumor immunity	Single-cell immune-metabolic profiling to separate tumor vs. immune cell effects. Engineered immune cells (CAR-T) with modulated PFK activity	Vu et al., 2022; Faial (2024), Du and Yang (2025)
Lack of real-time metabolic monitoring	Inability to track glycolytic inhibition dynamically in patients	Develop PFKFB3/4-specific PET tracers. Genetically encoded biosensors for F2,6BP in preclinical models	Rahar and Singh (2025)

Metabolic plasticity and resistance are quite challenging because cancer cells tend to switch energy sources and adopt alternative pathways when glycolysis is inhibited. In this review, we discussed how synthetic (e.g., PFK15 and metformin) and natural (e.g., betulinic acid and 5-FU) combination therapies represent a direct strategy for combating plasticity. Besides, future use of cutting-edge technology, such as synthetic lethality screens via CRISPR technology to determine the pathways of compensatory mechanisms and spatial metabolomics to map metabolic peri-tumor variations in real time. These approaches can be used to identify effective combination strategies, including PFK inhibitors combined with OXPHOS or glutaminolysis inhibitors (Cadassou and Jordheim, 2023; Wang H. et al., 2025; Karpe et al., 2026).

The identification of PFKP as a protein kinase, based on recent discoveries of its moonlighting functions in the nucleus, and of PFKFB3, which reveals previously unknown weaknesses (Chen et al., 2019; Gao et al., 2021; Deng et al., 2024; Wan et al., 2025; Liu et al., 2026). In addition to the classic components of glycolysis, PFKP can relocate to the nucleus to regulate gene expression, and PFKFB4 possesses intrinsic kinase activity that drives oncogenic signaling pathways, independent of its metabolic function. This review is also among the first to summarize these results and suggest that coming-generation inhibitors should target these non-canonical interactions, and not only their catalytic activity. This would allow selective targeting of cancer cells and may help preserve normal tissues.

The proposed approach of targeting PFK in TAMs and CAFs is a two-fold strategy: it inhibits lactate-driven immunosuppression while also risking impairment of the glycolytic metabolism necessary for T-cell function. PFKFB3 activity in TAMs and CAFs leads to lactate production and a decrease in pH within the TME, which promotes M2 polarization, and PD-L1 expression and suppresses the cytotoxic activity of CD8^+^ T cells (Chesney et al., 2016; Zhang et al., 2025). PFKFB3 inhibitors reduce lactate production, restore T-cell activity, and improve the effectiveness of anti-programmed cell death protein-1/PD-L1 checkpoint therapies in preclinical studies (Chen et al., 2019; Lu S. et al., 2024; Wang T.-T. et al., 2025; Zhang et al., 2025). However, since activated T cells rely on aerobic glycolysis for their growth and function, systemic inhibition of PFK could impair their ability to mount an effective anti-tumor response (Toledano Zur et al., 2024). Therefore, to achieve therapeutic success, strategies are needed to separate these effects, such as using isoform-specific inhibitors, delivering drugs locally to the stroma, or combining therapies that temporarily suppress stromal glycolysis while preserving T cell metabolic health (Wang T.-T. et al., 2025; Yuan et al., 2025; Zhang et al., 2025). Additionally, the TME influences treatment, since PFK isoforms expressed on stromal cells support immune evasion and resistance. PFK expression in tumor and stromal regions can be analyzed using techniques such as spatial transcriptomics or multiplex imaging, and stromal-targeted combination treatments can be tested in 3D organoid co-culture models described by Du and Yang (2025), Faial (2024), and Vu et al. (2022).

Despite previous studies highlighting the low bioavailability of natural PFK inhibitors. This review stresses the need to exploit modern nanodelivery tools as a key measure to bring these potentially effective compounds to the clinic. Therefore, addressing difficulties in delivering treatment and achieving specificity requires new systems and imaging methods. PFK-targeting ligands can be used as nanoparticle platforms to enhance tumor targeting. In contrast, PFK-targeted PET tracers can be used to track drug distribution and target interaction in a non-invasive manner. Such innovations will widen the meanings of therapy and reduce off-target effects (Rahar and Singh, 2025).

Integrated multi-omics, such as single-cell RNA sequencing, proteomics, and metabolomics, could help overcome the problem of biomarker deficiency in stratifying patients with PFK dependence by identifying predictive signatures. The machine learning models trained on such datasets may then be used to identify patients with the highest likelihood of deriving value from PFK-targeted therapies (Lu Z. et al., 2024; Shi et al., 2025). Modification of combination therapy regimens is an optimized procedure that needs advanced preclinical testing platforms.

Patient-derived organoid-based high-throughput drug screening enables rapid evaluation of a variety of drug combinations. Also, digital twins predict treatment response *in silico* before the clinical trial (Hernandez-Boussard et al., 2021; Stahlberg et al., 2022). The non-metabolic activities of the PFK isoforms, e.g., in transcription and DNA repair, contribute to the complexity of targeting therapeutically but provide new vulnerabilities. These unusual signaling networks can be discovered using such techniques as comprehensive phosphoproteomics and post-translational modification mapping. In the meantime, CRISPR-based functional genomics can be used to correlate certain isoform functions with phenotypic impacts (Shang et al., 2025; Xu D. et al., 2025). To successfully overcome resistance through compensatory pathways, researchers are recommended to use polypharmacology approaches, including multi-omics validation, and to monitor real-time alterations in metabolic flux using live-cell biosensors (Lu Z. et al., 2024; 2026).

To hasten clinical translation, biomarker-enriched trials and PFK-specific imaging assessment methods, including continuous pharmacodynamic PET, should be used in patients (Ogura et al., 2015; Xu Y. et al., 2025). In addition, to address cancer heterogeneity, it is necessary to create pan-cancer dependency maps via systematic CRISPR screening and to use AI-based classification to determine PFK-driving molecular subtypes across various malignancies (Menon et al., 2025). The impact on the immune cells is both a challenge and an opportunity. As much as PFK inhibition may undermine antitumor immunity, the same can be managed strategically. Immune-metabolic profiling of single cells helps identify effects on either the tumor or immune cells. Also, this could be enhanced by the engineering of CAR-T cells with modulated PFK activity to reduce their survival and efficacy in the TME (Si et al., 2026). Nevertheless, the lack of real-time metabolic monitoring in patients does not improve treatment optimization. Development of PFKFB3/4-specific PET tracers and biosensors for major glycolytic intermediates will enable real-time evaluation of treatment response and assist in adaptive therapy (Rahar and Singh, 2025). A combination of these sophisticated research instruments, including AI-based drug development, single-cell omics, spatial metabolomics, and digital twin modeling, will help the field address the current limitations of PFK-targeted cancer therapy. This broad-spectrum plan will accelerate the development of more desired specific inhibitors, identify reliable biomarkers, develop effective combination therapies, and eventually transform PFK-targeting agents into viable clinical therapies. The intended purpose of these strategies is to exploit cancer’s metabolic weaknesses and minimize damage to normal tissues.

To make further recommendations, we propose that the researchers consider targeting PFKFB3 with a basket trial design, which would be a rational, tumor-agnostic approach. This includes the enrolment of patients with different types of hematological and solid cancers having a high PFKFB3 expression or activity. Non-invasive functional metabolic imaging techniques can be used to perform effective patient stratification in such trials: 18F-FDG PET, which provides an image of glycolytic flux, and the measurement of circulating metabolic biomarkers, such as the lactate-to-pyruvate ratio as a surrogate marker of PFKFB3-driven glycolysis and an early pharmacodynamic indicator (Xu Y. et al., 2025). Also, the PFKFB3 inhibition in combination with immunotherapy is highly supported by the mechanism by which it decreases the production of lactate in the tumor and thereby lessens the immunosuppressive acidic TME, restores the cytotoxic T-cell function, and, possibly, transforms an immune-cold tumor into an inflamed tumor more susceptible to checkpoint inhibitors, such as anti-programmed cell death protein-1/PD-L1.

## 11 Conclusion

The pathophysiological basis of PFK is vital to cancer metabolism; therefore, this review explains the rationale for targeting it therapeutically. The PFK isoforms (PFKP, PFKM, and PFKL) and their primary allosteric modifiers, the PFKFB enzymes (particularly PFKFB3 and PFKFB4), are a major regulatory “hub” of glycolysis and play a pivotal role in cancer metabolism. PFK’s complexity is reflected in its tissue-specific isoforms, regulation of its oligomeric states, and its allosteric regulation by a range of metabolites, including F2,6BP, ATP, and citrate. This structural perspective grounds the role of mutations in the formation of the role, both in inherited diseases such as Tarui disease and in cancer mutations in the soma, in their contribution to abnormal glycolytic activity. While the ongoing discovery of new PFK inhibitors remains crucial, future progress will probably come from leveraging the deep biological understanding of PFK. This includes its isoform-specific regulation, allosteric mechanisms, and moonlighting functions, to systematically design and combine synthetic and natural inhibitors for tailored, patient-specific therapies.

PFKFB3 and PFKFB4, aside from their canonical roles in glycolysis, aid in cancer progression by enhancing cell proliferation, invasion, and metastasis through sustaining aerobic glycolysis and EMT. They also help address therapy resistance by promoting metabolic reprogramming, suppressing ferroptosis, and DNA repair. Within the TME, CAFs and TAMs expressing PFKFB3 facilitate metabolic symbiosis, immunosuppressive differentiation, and angiogenesis, thereby promoting tumor growth. Also, these enzymes have non-metabolic functions - such as nuclear PFKP regulating transcription and PFKFB4 as a protein kinase-that further expand their oncogenic potential beyond metabolism.

PFK has been of great therapeutic interest. The glycolytic activity can be reduced by both synthetic (e.g., PFK15, clotrimazole, and AICAR) and natural bioactive compounds (e.g., curcumin, resveratrol, and EGCG), which promote apoptosis and enhance the efficacy of conventional therapies. The potential use of PFK-based therapeutic strategies is supported by preclinical and early clinical trials, as well as by findings on the role of PFKFB3 in radiotherapy response and on PFKP methylation as a diagnostic biomarker.

However, several challenges and unmet needs remain. First, existing inhibitors are not isoform-selective, potentially leading to off-target effects and toxicity. Another challenge is the metabolic plasticity of cancer cells, driven by metabolic reprogramming, which enables rapid adaptation when glycolysis is pharmacologically targeted, with a shift toward alternate bioenergetic and biosynthetic pathways, such as OXPHOS and glutaminolysis, to sustain ATP production, redox state, and macromolecule synthesis. Such metabolic reprogramming has been observed with PFKFB3 inhibitors, where the inhibition leads to a switch to mitochondrial respiration and glutamine-dependent anaplerosis, ultimately decreasing therapeutic efficacy. Such metabolic flexibility not only drives resistance but also has implications for preclinical drug studies, which rely on simple *in vitro* models that lack the dynamic TME, which plays an important role in determining pathway availability. Also, many potential drugs, particularly natural products, often exhibit poor pharmacokinetics (e.g., low bioavailability and high metabolic instability), further reducing the time to glycolytic inhibition before alternative pathways are activated.

The research gaps include insufficient understanding of isoform-specific functions across different cancers, the impact of PFK inhibition on anti-tumor immune responses, and the identification of validated biomarkers to distinguish patients with highly glycolytic tumors. There is also a need to study mechanisms of resistance to PFK-targeted treatments and the structural specifics of interactions with the target’s inhibitor class, including auspicious ones such as PFK15.

To turn the mechanistic insights from this review into practical clinical applications, the following strategic priorities—based on the integrated Pillar 1–3 PFK-targeting ecosystem—must be addressed.

- *Isoform-selective inhibitors development:* AI-driven drug discovery, structural biology, and high-throughput screening can be used to develop compounds that selectively target cancer-related isoforms such as PFKFB3 and not normal tissue isoforms.
- *Rational combination therapies*: Developing synergistic treatment regimens that include PFK inhibitors with agents that act on other metabolic pathways (such as OXPHOS or glutaminolysis inhibitors), standard chemotherapy, radiotherapy, or immunotherapy, to overcome resistance and enhance activity.
- *Sophisticated translational models*: The use of patient-derived organoids, 3D co-culture models, and genetically engineered mouse models with improved tumor-stroma interactions and metabolic diversity to test clinical therapies.
- *Novel delivery systems*: Developing nanocarriers and ligand-targeted systems to improve bioavailability and tumor targeting and pharmacokinetics of PFK inhibitors, particularly of natural products.
- *Combination of multi-Omics and real-time monitoring*: Using spatial metabolomics, single cell sequencing, and PFK-specific PET tracers to identify predictive biomarkers, dynamic monitoring of metabolic changes in real-time, and dynamic monitoring of treatment response.
- *Target the immune-metabolic interface*: Understand the role of PFK modulation in altering immune cell activity in the tumor environment to come up with approaches that prevent tumor metabolism, but do not kill it, and perhaps enhance anti-tumor immunity, including checkpoint blockade therapy.

To conclude, PFK is an important nexus in cancer metabolism, signaling, and therapy resistance. The PFK-targeting ecosystem framework proposed herein underscores that the future of this field lies in the deliberate integration of deep biological understanding with a diverse therapeutic arsenal and robust clinical translation strategies. The combination of structural information, high-throughput screening of compounds, and combination therapy all point to a bright future for treating cancer through metabolism, despite its relative selectivity, plasticity, and translation issues. Interdisciplinary collaboration and new technologies are essential for developing efficient PFK-targeted interventions that may disrupt the metabolic underpinnings of cancer and improve patient outcomes in precision oncology.

## Glossary

ADP: Adenosine diphosphate
AKT: Protein kinase B
AMP: Adenosine monophosphate
AMPK: Adenosine monophosphate-activated protein kinase
ATP: Adenosine triphosphate
BC: Breast cancer
BRCA: Breast invasive carcinoma
CAFs: Cancer-associated fibroblasts
CESC: Cervical squamous cell carcinoma
CHOL: Cholangiocarcinoma
COAD: Colon adenocarcinoma
CRC: Colorectal cancer
DMAMC: Dimethylaminomicheliolide
EGCG: Epigallocatechin-3-gallate
EGF: Epidermal growth factor
EGFR: Epidermal growth factor receptor
EMT: Epithelial-mesenchymal transition
ESCA: Esophageal carcinoma
F2,6BP: Fructose-2,6-bisphosphate
FTO: Fat mass- and obesity-related gene
HER2: Human epidermal growth factor receptor-2
HIF-1*α*: Hypoxia inducible factor-1*α*
HK: Hexokinase
HRD1: HMG-CoA reductase degradation 1
KICH: Kidney chromophobe
KIRC: Kidney renal clear cell carcinoma
LDH: Lactate dehydrogenase
LIHC: Liver hepatocellular carcinoma
LUAD: Lung adenocarcinoma
mTOR: Mammalian target of rapamycin
NF-*κ*B: Nuclear factor kappa B
NSCLC: Non-small cell lung cancer
OXPHOS: Oxidative phosphorylation
PD-L1: Programmed death-ligand-1
PFK: Phosphofructokinase
PFKFB: 6-phosphofructo-2-kinase/fructose-2,6-bisphosphatases
PI3K: Phosphoinositide 3-kinase
PKM2: Pyruvate kinase M2
PKP1: Plakophilin 1
STAD: Stomach adenocarcinoma
TAMs: Tumor-associated macrophages
TME: Tumor microenvironment
