# Supplementary material for: Targeting phosphofructokinase in cancer: integrating natural products for metabolic reprogramming and therapeutic innovation
Source: Front Pharmacol. 2026 Jun 10;17:1844992. doi: 10.3389/fphar.2026.1844992 (PMC13291013; doi:10.3389/fphar.2026.1844992)
Supplement: Supplementary file 1 [file Supplementaryfile1.docx]

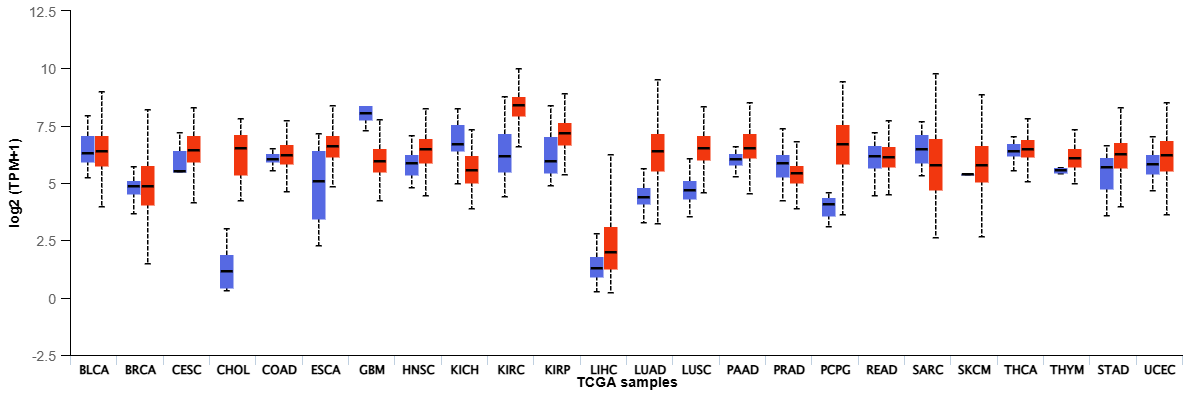


**Supplementary Figure 1**. Expression of phosphofructokinase platelet type (PFKP) in tumor and normal samples retrieved from TCGA (<https://ualcan.path.uab.edu/analysis.html>) datasets.


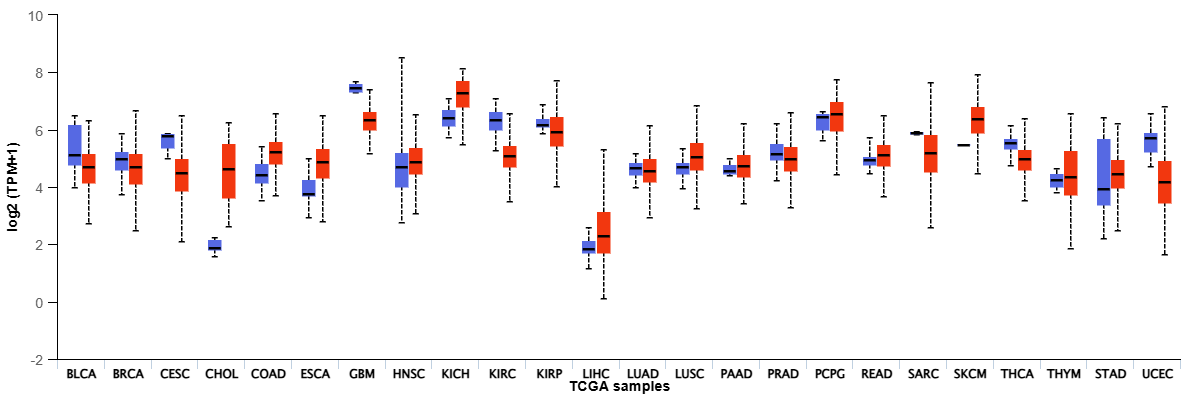


**Supplementary Figure 2**. Expression of phosphofructokinase muscle type (PFKM) in tumor and normal samples retrieved from TCGA (<https://ualcan.path.uab.edu/analysis.html>) datasets.


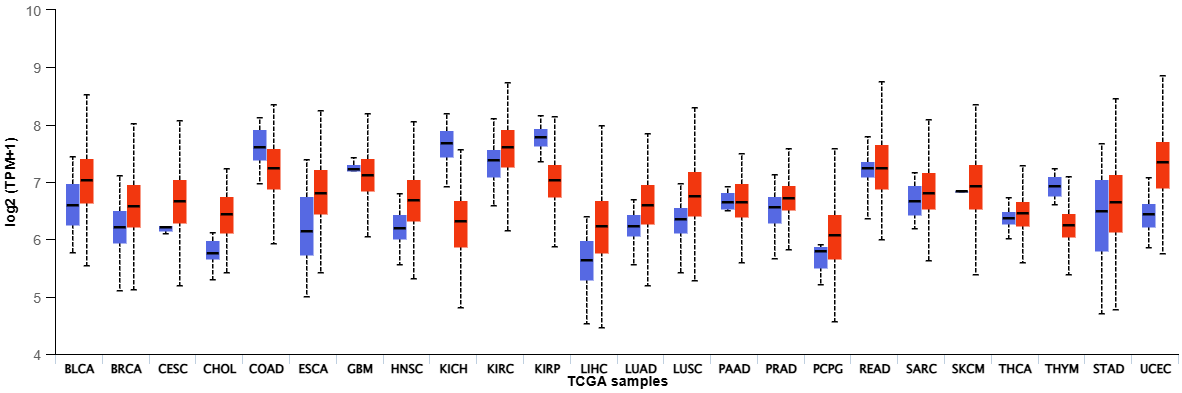


**Supplementary Figure 3**. Expression of phosphofructokinase liver type (PFKL) in tumor and normal samples retrieved from TCGA (<https://ualcan.path.uab.edu/analysis.html>) datasets.
